# Supplementary material for: Emergent honeycomb network of topological excitations in correlated charge density wave
Source: Nat Commun. 2019 Sep 6;10:4038. doi: 10.1038/s41467-019-11981-5 (PMC6731227; doi:10.1038/s41467-019-11981-5)
Supplement: Supplementary file 1 — Supplementary Information [file 41467_2019_11981_MOESM1_ESM.pdf]

Emergent Honeycomb Network of Topological Excitations in Correlated Charge Density Wave

Park et al.

Supplementary Note 1: STS spectra and DFT calculations

We provide additional information about the STS spectra, Mott states of the theoretical models and interlayer coupling effect.

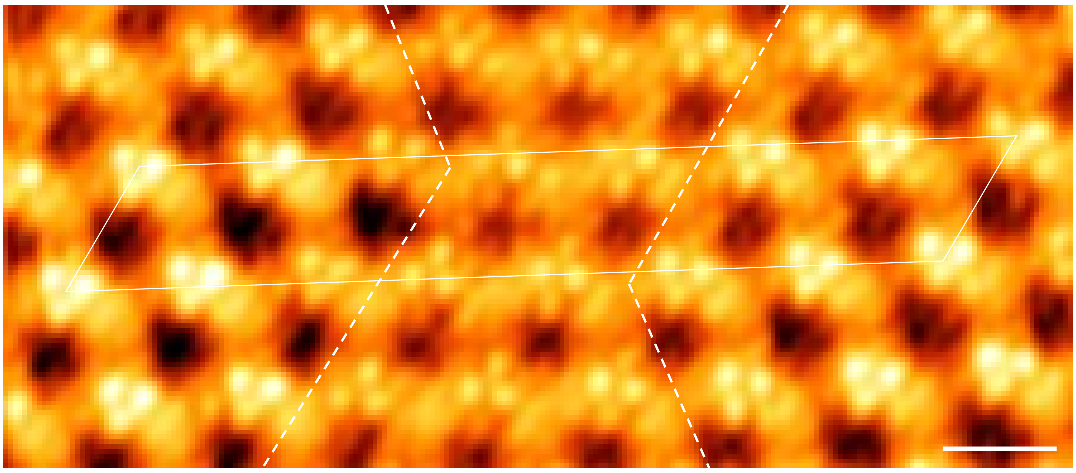

Supplementary Fig. 1: Well filtered STM image of the NC phase. Dashed lines separate two different domains and domain wall. The solid lines represent the same area as Fig. 2c. *Scale bar*, 1 nm.

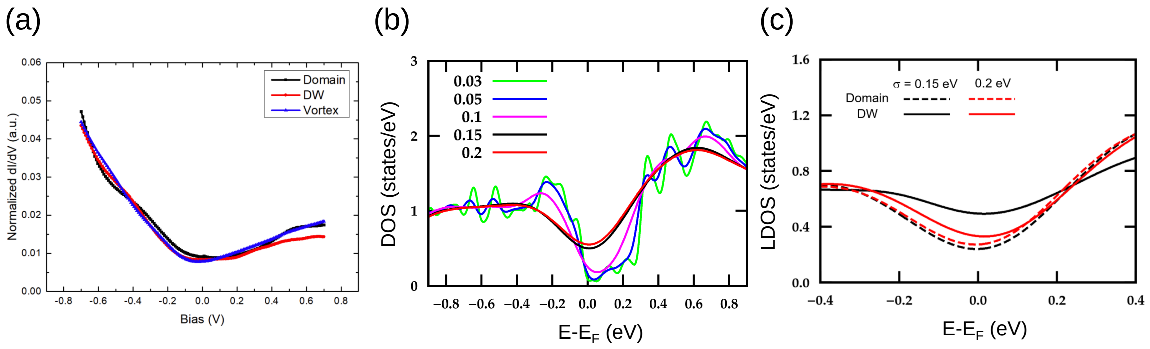

Supplementary Fig. 2: STS spectra of the NC phase and theoretical DOS of DW-1 structure. (a) STS spectra, (b) Total DOS as a function of the electronic temperature parameter σ (eV). (c) Local DOS of domain and domain wall regions ($\sigma = 0.15$ and 0.2 eV).

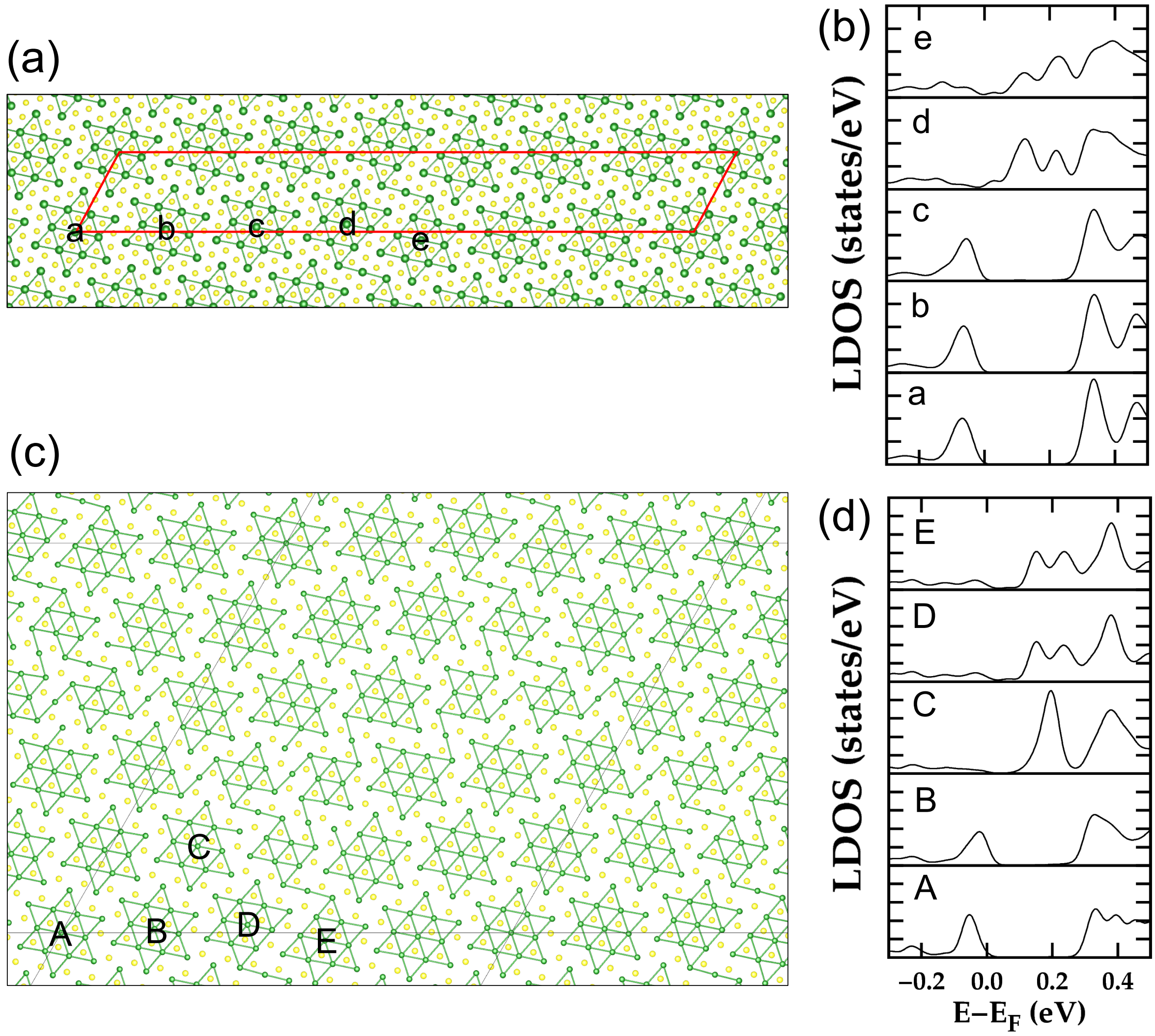

Supplementary Fig. 3: Mott states in domain and domain wall. (a) Atomic structure of the DW-1 and (b) Local DOS at center Ta atom of David stars. (c) Atomic sturcture of the hexagonal domain model. (d) Local DOS.

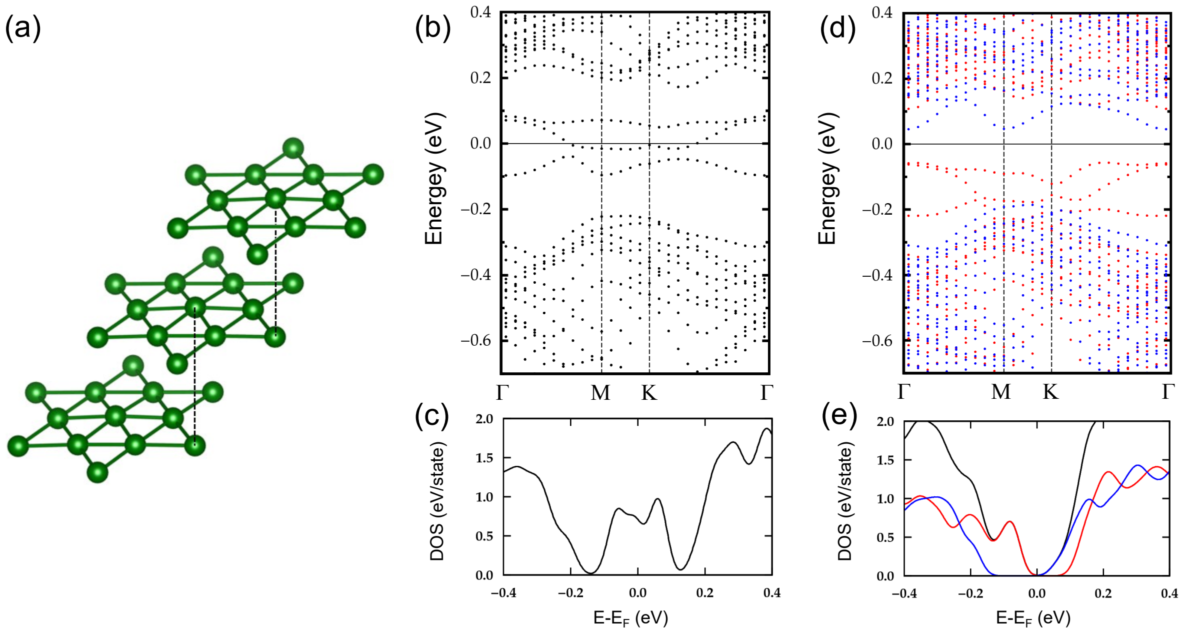

Supplementary Fig. 4: One possible tri-layer stacking order in the C phase. (a) atomic structure, (b) band structure and (c) total DOS without electron-electron correlations. (d) band structure and (e) total DOS with electron-electron correlations. The interlayer distance of 5.9 Å is fixed.

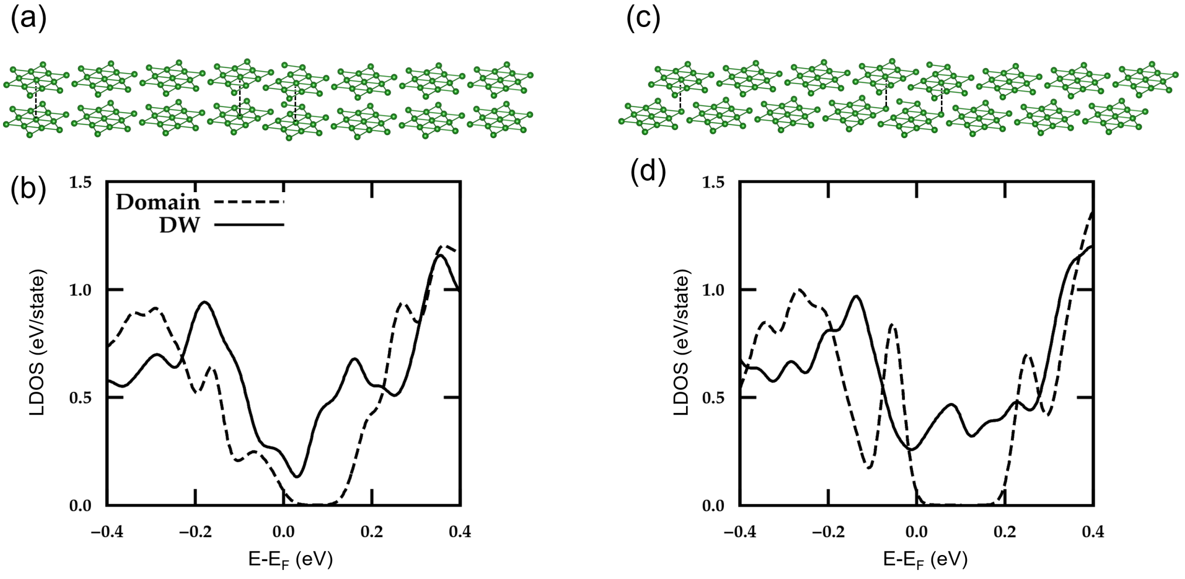

Supplementary Fig. 5: Two different bilayer stacking order in the DW-1 structure. (a) Atomic structure of the on-top stacking and (b) Local DOS at domain and domain wall. (C) Atomic structure of the shifted staking and (d) Local DOS at domain and domain wall. All atoms are fixed at their atomic position in the single-layer DW-1 structure. The interlayer distance of 5.9 Å is fixed.

Supplementary Note 2: Construction of Network Model

In this supplementary material, we present the details of the honeycomb network model. Here we apply the strategy of Ref. [1] to the honeycomb lattice.

We theoretically model the system by a regular array of one-dimensional metals living on the links of a honeycomb lattice. The construction of this model is motivated from the following experimental observations present in the main text.

1. **Emergent honeycomb lattice**: The domains of the NC-CDW state form a regular honeycomb lattice, and the domain walls are the links of this honeycomb lattice.

2. **Metallic domain walls**: The domain walls trap finite local density of states near the Fermi level (Note that this is generically expected for any domain wall of a charge-density wave since the domain wall carries in-gap states whose origin are topological [2]).

Motivated from these, we consider a regular array of one-dimensional metals living on the links of a honeycomb lattice. Similar network models of one-dimensional metals have been studied to some degree in the context of quantum Hall plateau transitions, known as "Chalker-Coddington model" [3], and recently have been revived to explain the physics of the twisted bilayer graphene at a small twisting angle [1]. We apply this latest theoretical progress to model the network on the honeycomb lattice.

Some details of the model are following:

1. **Degrees of freedom**: On each link $a = x, y, z$ of the honeycomb lattice, we assign the two wavefunctions ψ_a and $\psi_{\bar{a}}$. Here ψ_a represent the chiral mode propagating from an A-sublattice to its neighboring B-sublattice and $\psi_{\bar{a}}$ for the mode propagating from a B-sublattice to its neighboring A-sublattice (See Fig. 6). Microscopically they correspond to the low-energy modes near the Fermi momentum of one-dimensional metals propagating along the links. (Here we suppress the spin index for the modes because we are mainly interested in the spectral properties of the network model.)

2. **Scattering between wavefunctions**: We assume that the modes propagate coherently within each link and scatter only at the nodes of the honeycomb lattice. We further assume that there are three-fold rotation and two-fold mirror symmetries at each nodes, and the scattering between the modes respects the crystal symmetries.

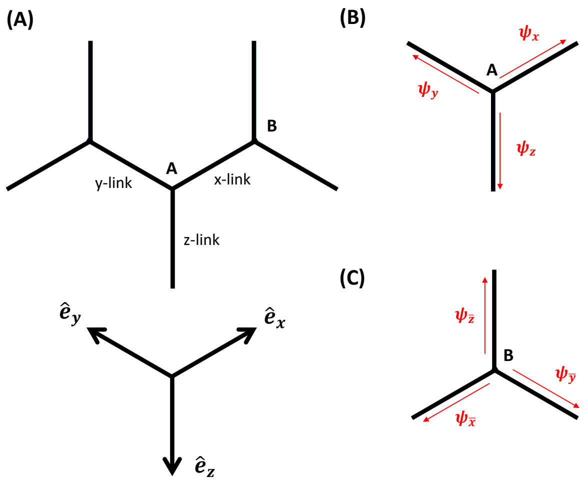

Supplementary Fig. 6: Pictorial Representation of Network Model: (A) labeling of the links by x, y, z on real space. Each link has the two degrees of freedom. One is from the neighboring A-sublattice [See (B)] and another is from the neighboring B-sublattice [See (C)]. They are written as ψ_a and $\psi_{\bar{a}}$, and the arrows represent the propagating directions of the modes. Here $\hat{e}_a, a = x, y, z$ is the vector connecting the A-sublattice to the B-sublattice.

Having these in mind, we have the following scattering problem at the A-sublattice.

$$\begin{bmatrix} \psi_x(\mathbf{R}) \\ \psi_y(\mathbf{R}) \\ \psi_z(\mathbf{R}) \end{bmatrix} = e^{-i\frac{E}{v_F\hbar}L} \cdot \hat{T}_A \cdot \begin{bmatrix} \psi_{\bar{x}}(\mathbf{R}+\hat{\mathbf{e}}_\mathbf{x}) \\ \psi_{\bar{y}}(\mathbf{R}+\hat{\mathbf{e}}_\mathbf{y}) \\ \psi_{\bar{z}}(\mathbf{R}+\hat{\mathbf{e}}_\mathbf{z}) \end{bmatrix} \tag{1}$$

Here, the left-hand side $\psi_a(\mathbf{R}), a = x, y, z$ represents the out-going modes from the A-sublattice, which is related by a scattering matrix $\hat{T}_A$ to the in-coming modes $\psi_{\bar{a}}(\mathbf{R}), a = x, y, z$ appearing on the right-hand side (See Fig. 6). The additional phase factor $\sim \exp(-i\frac{E}{v_F\hbar}L)$ is the phase accumulated by the incoming modes while it propagates coherently from the neighboring B-sublattices to the A-sublattice at $\mathbf{R}$. Here v_F is the Fermi velocity within the one-dimensional metal, which is expected to be similar to that of the bulk electron, and L is the length of the link. The scattering matrix $\hat{T}_A$ can be fixed by the three-fold rotation as well as the two-fold mirrors at the node. With the unitarity of the scattering matrix, we find

$$\hat{T}_A = e^{i\chi_A} \begin{bmatrix} T_A & t_A & t_A \\ t_A & T_A & t_A \\ t_A & t_A & T_A \end{bmatrix}, \quad |T_A| \in \left[\frac{1}{3},\, 1\right],\ t_A = e^{i\phi_A}\sqrt{\frac{1-|T_A|^2}{2}}, \tag{2}$$

with $\phi_A = \cos^{-1}(\frac{|t_A|}{2|T_A|})$. Similarly we have the following scattering problem at the B-sublattice.

$$\begin{bmatrix} \psi_{\bar{x}}(\mathbf{R}) \\ \psi_{\bar{y}}(\mathbf{R}) \\ \psi_{\bar{z}}(\mathbf{R}) \end{bmatrix} = e^{-i\frac{E}{v_F\hbar}L} \cdot \hat{T}_B \cdot \begin{bmatrix} \psi_x(\mathbf{R}-\hat{\mathbf{e}}_\mathbf{x}) \\ \psi_y(\mathbf{R}-\hat{\mathbf{e}}_\mathbf{y}) \\ \psi_z(\mathbf{R}-\hat{\mathbf{e}}_\mathbf{z}) \end{bmatrix}, \tag{3}$$

where $\hat{T}_B$ has the same structure as the $\hat{T}_A$.

Now we can perform the Fourier transformation on $\mathbf{R}$ and solve these scattering problems.

$$\mathbf{\Psi}_\mathbf{q} = e^{-i\frac{E_\mathbf{q}}{v_F\hbar}L}\hat{T}_\mathbf{q}\cdot\mathbf{\Psi}_\mathbf{q}, \quad \mathbf{\Psi}_\mathbf{q} = \begin{bmatrix} \psi_x(\mathbf{q}) \\ \psi_y(\mathbf{q}) \\ \psi_z(\mathbf{q}) \\ \psi_{\bar{x}}(\mathbf{q}) \\ \psi_{\bar{y}}(\mathbf{q}) \\ \psi_{\bar{z}}(\mathbf{q}) \end{bmatrix}, \quad \hat{T}_\mathbf{q} = \begin{bmatrix} 0 & \hat{T}_A\cdot\hat{V}_\mathbf{q} \\ \hat{T}_B\cdot\hat{V}^*_\mathbf{q} & 0 \end{bmatrix}, \tag{4}$$

where $\hat{V}_\mathbf{q} = \text{diag}\ [\exp(i\mathbf{q}\cdot\hat{\mathbf{e}}_\mathbf{x}),\ \exp(i\mathbf{q}\cdot\hat{\mathbf{e}}_\mathbf{y}),\ \exp(i\mathbf{q}\cdot\hat{\mathbf{e}}_\mathbf{z})]$. Hence, the energy spectrum can be obtained by diagonalizing $\hat{T}_\mathbf{q}$, which is again an unitary matrix. In terms of the eigenvalues $e^{i\epsilon_j(\mathbf{q})}, j = 1, 2, \cdots 6$ of $\hat{T}_\mathbf{q}$, we have the energy spectrum:

$$E^n_{j,\mathbf{q}} = 2\pi\frac{v_F\hbar}{L}n + \frac{v_F\hbar}{L}\epsilon_j(\mathbf{q}), \quad j = 1, 2, \cdots 6. \tag{5}$$

Here $n \in \mathbb{Z}$ and thus the minibands are repeating in the energy in period of $2\pi\frac{v_F\hbar}{L}$. Mathematically this repetition in n originates from the ambiguity of $\epsilon_j(\mathbf{q})$ by 2π appearing in the eigenvalues $e^{i\epsilon_j(\mathbf{q})}, j = 1, 2, \cdots 6$. Physically this repetition can be traced back to the excitation energy of the microscopic one-dimensional modes with the same momentum $\mathbf{q}$, i.e., for a given $\mathbf{q}$, there are multiple different one-dimensional modes with energy $2\pi\frac{v_F\hbar}{L}n,\ n \in \mathbb{Z}$. Thus we expect that the energy spectrum given by $\frac{v_F\hbar}{L}\epsilon_j(\mathbf{q})$ will repeat in energy with a period $2\pi\frac{v_F\hbar}{L}$ and entirely fills up the bulk CDW gap. Below we will analyze only one period of the band spectrum.

Supplementary Note 3: Band Spectrum

We first consider the case where we have a full symmetry of the honeycomb lattice, i.e., $\hat{T}_A = \hat{T}_B$. As apparent from the Fig 7, the spectrum features (i) Dirac cones at the K and K' points, (ii) flat bands, and (iii) quadratic band touchings at the Γ point. Now we discuss the stabilities of these features.

1. **Dirac Cones at the K and K' points**: The Dirac cones are protected by the sublattice symmetry as in the graphene. It is easily removed by breaking the symmetry, i.e., $T_A \neq T_B$. See the spectrum in Fig 7.

2. **Quadratic Band Touching at the Γ point**: The quadratic band touchings can be protected by the six-fold rotation symmetry [4]. However, even when the symmetry is broken (while keeping the three-fold rotation and mirror symmetries are kept), the band touchings are robust within our network model. See the Fig 7.

3. **"Flat-ness" of Flat bands**: The flat-ness of the bands cannot be protected. However, within our network model (with the three-fold rotation C_3 and mirror symmetries), we find that it is robust. See the Fig 7.

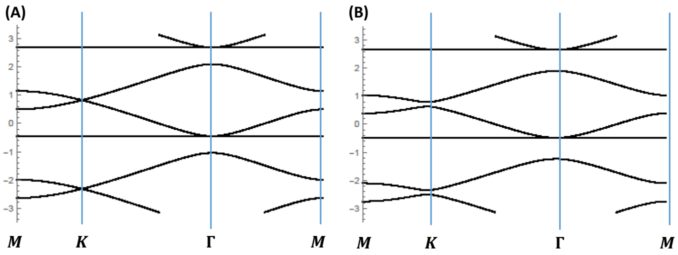

Supplementary Fig. 7: Energy Spectrum of Honeycomb Network Model. Here we plot the spectrum of $\epsilon_j(\mathbf{q})$ of the scattering matrix $\hat{T}_{\mathbf{q}}$ along $M \to K \to \Gamma \to M$. (A) C_6-symmetric spectrum $\hat{T}_A = \hat{T}_B$. It is straightforward to note the Dirac fermion at the K point, quadratic band touching at Γ point, and also the flat bands. (B) C_3-symmetric spectrum $\hat{T}_A \neq \hat{T}_B$. Here the Dirac band touching is removed.

Supplementary Note 4: Symmetry Analysis of Quadratic Band Touching

Here we perform the symmetry analysis of the quadratic band touchings at the Γ point. By explicitly diagonalizing Eq.(4) at the Γ point (with $\hat{T}_A = \hat{T}_B$), we obtain the wavefunctions of the degenerate states. They are labeled as $|\Psi_a\rangle, a = 1, 2$ (with $\langle\Psi_a|\Psi_b\rangle = \delta_{ab}$), from which we reconstruct the representations of the three-fold rotation C_3, the x-mirror $R_x : x \to -x$, and the six-fold rotation C_6, i.e., $\{[C_3]_p, [R_x]_p, [C_6]_p\}$ within these bands. For example, we obtain the three-fold rotation $[C_3]_p$ within the two states by computing

$$[C_3]_p = \begin{bmatrix} \langle\Psi_1|\hat{C}_3|\Psi_1\rangle & \langle\Psi_2|\hat{C}_3|\Psi_1\rangle \\ \langle\Psi_1|\hat{C}_3|\Psi_2\rangle & \langle\Psi_2|\hat{C}_3|\Psi_2\rangle \end{bmatrix}, \tag{6}$$

in which $\hat{C}_3$ is the representation of the three-fold rotation in the six-component $\Psi_{\mathbf{q}}$ in Eq.(4).

By computing these explicitly, we find

$$[C_3]_p = e^{\frac{2\pi i}{3}\sigma^z}, \ [R_x]_p = e^{-\frac{\pi i}{3}\sigma^z}\sigma^x, \ [C_6]_p = e^{-\frac{2\pi i}{3}\sigma^z}, \tag{7}$$

where $\sigma^a, a = x, y, z$ is the Pauli matrix acting on the space spanned by $\{|\Psi_1\rangle, |\Psi_2\rangle\}$.

With these, we can write down symmetry-allowed Hamiltonian near the Γ point. First, by imposing $[C_3]_p$ and $[R_x]_p$, we find that no perturbation is allowed to split $|\Psi_1\rangle$ and $|\Psi_2\rangle$ at the Γ point.

$$[C_3]_p^\dagger H_0 [C_3]_p = H_0, \ \ [R_x]_p^\dagger H_0 [R_x]_p = H_0, \ \ \to H_0 \propto \mu\sigma^0 \tag{8}$$

Hence the degeneracy cannot be removed when $[C_3]_p$ and $[R_x]_p$ are imposed. On the other hand, near the Γ point, we find that the linear band touching is allowed.

$$[C_3]_p^\dagger H(\mathbf{k})[C_3]_p = H(C_3^{-1}[\mathbf{k}]), \ \ [R_x]_p^\dagger H(k_x, k_y)[R_x]_p = H(-k_x, k_y), \tag{9}$$

allows $H(\mathbf{k}) \propto k_x\hat{s}^x + k_y\hat{s}^y$ (where $(\hat{s}_x, \hat{s}_y)$ are the Pauli matrices obtained by properly rotating σ^x and σ^y.). Hence, the quadratic band touching cannot be protected by $[C_3]_p$ and $[R_x]_p$. Nevertheless, within our network model, the touching is found to be robust though the touching is not protected by the symmetries.

We can show that we need the six-fold rotation symmetry $[C_6]_p$ to protect the quadratic band touching and this is consistent with Ref. [4]. Thus, on imposing $[C_6]_p$, we can fix the Hamiltonian as

$$H = \epsilon_0(|\mathbf{k}|) + \Big(\frac{k_x^2 - k_y^2}{2m}\sigma^x + \frac{2k_xk_y}{2m}\sigma^y\Big). \tag{10}$$

To match the band spectrum seen in the model, we have $\epsilon_0(|\mathbf{k}|) = \frac{\mathbf{k}^2}{2m}$ and thus the lower band is completely flat and the density of state at the zero energy is divergent.

Supplementary Note 5: Comparison with Twisted Graphene Bilayer

Here we extend our discussion in the main text on the similarity between our network system and the theoretical models [1, 5] for the twisted graphene bilayers. In particular, we compare ours with the network model in Ref. [1] and a continuum Dirac fermion model in Ref. [5].

To start with, we find that our network system is close to the network model appeared in Ref [1]. In Ref. [1], the twisted graphene bilayer at a small twisting angle has been considered. When the twisting angle is small, there is a periodic array of domain walls separating the locally AA-stacked regions and the locally AB-stacked regions. Ref. [1] argued that these domain walls trap localized one-dimensional metallic channels. These one-dimensional modes scatter at the nodes, which form a triangular lattice (in our case, the nodes form a honeycomb lattice). The structure of their model is quite similar to ours and indeed Ref. [1] obtained a similar spectrum as ours: Dirac fermions, nearly flat bands, as well as van-Hove singularities.

We can also make a comparison of our network system with the continuum Dirac theory of magic-angle twisted bilayer graphene in Ref. [5]. In this approach, the Dirac fermions coming from the top and bottom layers interfere each other, and as a result, the bands become flat. Theoretically, this flat spectrum is speculated to be the source of surprising correlation-driven phenomena seen in the experiments [6–8]. One may note that our network model appears to be different than the continuum Dirac theory of Ref. [5]. Despite of the difference in the theoretical treatments, we emphasize that our network model and the result of Ref. [5] share the strikingly-similar features in spectrum: Dirac fermions, flat bands and associated singularities in density of states, which are believed to play an essential role in the correlation physics.

Both the twisted bilayer graphene and our honeycomb network have weak disorders [9, 10]. For example, there are some imperfect hexagons in our network and imperfect triangles in twisted bilayer graphene. Naively one expects that such weak disorders would immediately localize the flat bands and completely destroy associated many-body physics. However, the previous study [11] surprisingly found that the flat bands do not get immediately localized but become critical. This implies that the flat bands are stronger against disorders than we naively expect. Though a more thorough investigation is desirable, we expect from the reference [11] that the flat bands retain relatively flat spectrum even with the weak disorders and hence is expected to remain very susceptible to many-body physics.

In summary, we have shown that the two systems, twisted graphene bilayer and our network system, share the surprising similarities including the flat bands and a large density of states, which are the key to the exotic correlation-driven phenomena.

Supplementary Note 6: Interlayer Coupling

In this subsection, we consider the effect of interlayer coupling to the electronic structures in the conducting network, and we will argue that, in general, the interlayer couplings between the layers will little affect the emergent electronic structures.

For the concrete-ness of our theoretical discussion, we first assume that the charge-density wave domains in the nearly commensurate phase remain insulating even after the inclusions of interlayer couplings [see SFig.5]. With this in hand, all the lowest-energy electronic states are in the domain walls in the conducting networks, and the interlayer couplings will introduce the coupling between these metallic modes inside the conducting networks living in different layers.

Among various possible couplings, the most important coupling, which can largely modify the band structure, is the electron hopping process between the layers. Note that this is proportional to the wavefunction overlap between the states of domain walls in different layers, and the states are highly localized within each domain walls. Hence, the effect of coupling will be strongly suppressed if not the networks are almost exactly overlapping to each other when seen from c-axis. From the available literature [12], we note that the networks in different layers are not correlated to each other and thus we expect that the emergent band structure of the low-energy theory will not be affected much by the interlayer coupling.

Supplementary References

[1] D. K. Efimkin and A. H. MacDonald, Helical network model for twisted bilayer graphene, Phys. Rev. B **98**, 035404 (2018).
[2] W. P. Su, J. R. Schrieffer, and A. J. Heeger, Solitons in Polyacetylene, Phys. Rev. Lett. **42**, 1698 (1979).

[3] J. T. Chalker and P. D. Coddington, Percolation, quantum tunnelling and the integer Hall effect, J. Phys. C: Solid State Phys. **21**, 2665 (1988).
[4] K. Sun, H. Yao, E. Fradkin, and S. A. Kivelson, Topological insulators and nematic phases from spontaneous symmetry breaking in 2D Fermi systems with a quadratic band crossing, Phys. Rev. Lett. **103**, 046811 (2009).
[5] R. Bistritzer and A. H. MacDonald, Moiré bands in twisted double-layer graphene, Proc. Natl. Acad. Sci. **108**, 12233 (2011).
[6] Y. Cao, V. Fatemi, S. Fang, K. Watanabe, T. Taniguchi, E. Kaxiras, and P. Jarillo-Herrero, Unconventional superconductivity in magic-angle graphene superlattices, Nature **556**, 43 (2018).
[7] B. Lian, Z. Wang, B. A. Berneving, Twisted Bilayer Graphene: A Phonon Driven Superconductor, ArXiv e-print (2018), arXiv:1807.04382 [cond-mat.mes-hall].
[8] H. C. Po, L. Zou, A. Vishwanath, and T. Senthil, Origin of Mott insulating behavior and superconductivity in twisted bilayer graphene, ArXiv e-print (2018), arXiv:1803.09742 [cond-mat.str-el].
[9] I. Brihuega, P. Mallet, H. González-Herrero, G. Trambly de Laissardiére, M. M. Ugeda, L. Magaud, J. M. Gómez-Rodríguez,1 F. Ynduráin, and J.-Y. Veuillen, Unraveling the intrinsic and robust nature of van Hove singularities in twisted bilayer graphene by scanning tunneling microscopy and theoretical analysis, Phys. Rev. Lett. **109**, 196802 (2012).
[10] I. Razado-Colambo, J. Avila, J.-P. Nys, C. Chen, X. Wallart, M.-C. Asensio, and D. Vignaud, NanoARPES of twisted bilayer graphene on SiC: absence of velocity renormalization for small angles Sci. Ref. **6**, 27261 (2016).
[11] J. T. Chalker, T. S. Pickles, and Pragya Shukla, Anderson localization in tight-binding models with flat bands, Phys. Rev. B **82**, 104209 (2010).
[12] D. Cho, S. Cheon, K.-S. Kim, S.-H. Lee, Y.-H. Cho, S.-W. Cheong, and H. W. Yeom, Nanoscale manipulation of the Mott insulating state coupled to charge order in 1T-TaS_2, Nat. Commun. **7**, 10453 (2016).
